# Mr. Purton, on the Croup

**Published:** 1800-03

**Authors:** T. Purton

**Affiliations:** Alcester


### *To the Editors of the Medical and Phyſical Journal.*

GENTLEMEN,

ON peruſing a letter, inſerted in the XIth Number of your Medical and Phyſical Journal, ſigned A. HUGGAN, wherein that gentleman objects to the practice of bleeding, in the Croup, I thought it incumbent upon me, among many others who have had much experience in that diſeaſe, to offer ſome remarks upon it.

As I have already laid before the public my plan of treatment in the Croup, I ſhall confine myſelf to that part wherein Dr. HUGGAN's practice ſo ſtrenuouſly oppoſes mine.*

He tells us, that his experience authorizes him to ſay, that opium, in the form of tincture, will, if in a doſe proportionate to the violence of the diſeaſe, give relief as ſpeedily as venæſection, or any other remedy. He afterwards acknowledges, that his practice in this diſeaſe has not been very conſiderable; but from the little that has fallen within his own obſervation, he cannot too forcibly deprecate the uſe of the lancet.

The ſucceſsful caſes which fell under this gentleman's care, muſt certainly (in my opinion) have been merely a ſpaſmodic affection of the muſcles of the glottis, that may, no doubt, give way to the uſe of opium; but, ſurely, it never could have been that ſtate of the diſeaſe, termed Cynanche Trachealis; as in that caſe, unleſs a general depletion had preceded its introduction, an aggravation of the ſymptoms, rather than a diminution, would, more probably, have been the conſequence.

I have had a number of ſucceſsful caſes of genuine or inflamatory Croup, in all of which, bleeding by leeches has been my principal dependance; and the greater part of them, I am very well aſſured, would have terminated fatally, if ſuch a plan had not

* Medical and Phyſical Journal, Vol. I. p. 510.

not been vigorouſly purſued. For the truth of this aſſertion, I can claim the ſupport of men of the firſt eminence in their profeſſion. But I wiſh it to be underſtood, that although my chief dependence is on bleeding, I never omitted the other uſual antiphlogiſtic remedies. In that ſtate of the diſeaſe, termed Spaſmodic Croup, I have frequently found that a gentle emetic, and the uſe of the warm-bath, have removed every bad ſymptom.

There is not one poſſible thing, in my opinion, which can depreciate the ſcience of medicine to a greater degree (with all deſcriptions of people), than where profeſſional men are ſo continually differing with and oppoſing each other in their practice; and ſuch is the preſent enlightened age, that there are few men who do not make the ſcience of the healing art a part of their ſtudies:—How great, then, muſt be their ſurprize, to ſee that even thoſe eſtabliſhed points, which have ſtood the teſt and experience of ages, and now continue as firmly eſtabliſhed as ever, cannot eſcape the attack of ſome of the profeſſion!

If you are of opinion, Gentlemen, that the facts and obſervations which I have made, may tend to eſtabliſh a more accurate idea of the Modus Curandi of this very formidable diſeaſe, for thoſe practitioners who may not have had many opportunities of meeting with it, you will oblige me by introducing them in a future Number of your uſeful publication. I am, Gentlemen,

Very reſpectfully,

Your humble ſervant,

